# ESCARGOT: an AI agent leveraging large language models, dynamic graph of thoughts, and biomedical knowledge graphs for enhanced reasoning

**DOI:** 10.1093/bioinformatics/btaf031

**Published:** 2025-01-22

**Authors:** Nicholas Matsumoto, Hyunjun Choi, Jay Moran, Miguel E Hernandez, Mythreye Venkatesan, Xi Li, Jui-Hsuan Chang, Paul Wang, Jason H Moore

**Affiliations:** Department of Computational Biomedicine, Center for Artificial Intelligence Research and Education, Cedars Sinai Medical Center, West Hollywood, CA 90069, United States; Department of Computational Biomedicine, Center for Artificial Intelligence Research and Education, Cedars Sinai Medical Center, West Hollywood, CA 90069, United States; Department of Computational Biomedicine, Center for Artificial Intelligence Research and Education, Cedars Sinai Medical Center, West Hollywood, CA 90069, United States; Department of Computational Biomedicine, Center for Artificial Intelligence Research and Education, Cedars Sinai Medical Center, West Hollywood, CA 90069, United States; Department of Computational Biomedicine, Center for Artificial Intelligence Research and Education, Cedars Sinai Medical Center, West Hollywood, CA 90069, United States; Department of Computational Biomedicine, Center for Artificial Intelligence Research and Education, Cedars Sinai Medical Center, West Hollywood, CA 90069, United States; Department of Computational Biomedicine, Center for Artificial Intelligence Research and Education, Cedars Sinai Medical Center, West Hollywood, CA 90069, United States; Department of Computational Biomedicine, Center for Artificial Intelligence Research and Education, Cedars Sinai Medical Center, West Hollywood, CA 90069, United States; Department of Computational Biomedicine, Center for Artificial Intelligence Research and Education, Cedars Sinai Medical Center, West Hollywood, CA 90069, United States

## Abstract

**Motivation:**

LLMs like GPT-4, despite their advancements, often produce hallucinations and struggle with integrating external knowledge effectively. While Retrieval-Augmented Generation (RAG) attempts to address this by incorporating external information, it faces significant challenges such as context length limitations and imprecise vector similarity search. ESCARGOT aims to overcome these issues by combining LLMs with a dynamic Graph of Thoughts and biomedical knowledge graphs, improving output reliability, and reducing hallucinations.

**Result:**

ESCARGOT significantly outperforms industry-standard RAG methods, particularly in open-ended questions that demand high precision. ESCARGOT also offers greater transparency in its reasoning process, allowing for the vetting of both code and knowledge requests, in contrast to the black-box nature of LLM-only or RAG-based approaches.

**Availability and implementation:**

ESCARGOT is available as a pip package and on GitHub at: https://github.com/EpistasisLab/ESCARGOT.

## 1 Introduction

Large Language Models (LLMs), such as GPT-4, have revolutionized natural language processing by demonstrating advanced capabilities in understanding and generating human-like text (Kojima *et al.* 2024). However, despite their proficiency, LLMs often produce incorrect or nonsensical outputs, commonly referred to as hallucinations, while still exhibiting strong logical reasoning abilities (Ji *et al.* 2023). In parallel to advancements in generative AI, the field of knowledge aggregation has been maturing, particularly through the development of knowledge graphs. As LLMs lack internal mechanisms to mitigate hallucinations, we propose ESCARGOT (Enhanced Strategy and Cypher-driven Analysis and Reasoning using Graph Of Thoughts), a novel Python library and AI agent designed to autonomously reason, adapt, and enhance decision-making processes by integrating the outputs of knowledge graphs with LLMs. ESCARGOT combines the reasoning power of LLMs with a dynamic, Python-executable Graph of Thoughts (GoT), and the factual accuracy of knowledge graphs. This is accomplished by enabling the agent to query a knowledge graph database via Cypher or utilize a vector database of the knowledge graph for contextual augmentation (Hong *et al.* 2023).

Current approaches to mitigating hallucinations include fine-tuning, post-processing verification steps, and constrained generation techniques (Matsumoto *et al.* 2024). However, these methods often fall short in complex reasoning scenarios, where a more advanced, strategic approach is required. Retrieval-Augmented Generation (RAG) is a popular approach that seeks to address these issues by integrating external information sources (Lewis *et al.* 2020). Despite its advantages, RAG has significant shortcomings, including limitations related to context length and the challenges associated with the often imprecise use of vector similarity search (Kandpal *et al.* 2023).

GoT frameworks have shown promise in organizing reasoning processes, surpassing Chain-of-Thoughts and Tree-of-Thoughts implementations (Besta *et al.* 2024). However, current GoT implementations are typically static and lack the adaptability needed for diverse and dynamic queries. ESCARGOT overcomes these limitations by dynamically generating a GoT based on the LLM’s initial strategy formulation and autonomously executing it through a robust knowledge extraction mechanism.

A key component of ESCARGOT is its use of Cypher queries on a knowledge graph. Knowledge graphs are powerful tools that structure and interconnect information, representing entities and their relationships. By enabling the GoT to query a knowledge graph using Cypher, ESCARGOT leverages the graph’s rich semantic relationships and logical structure to retrieve more accurate and contextually relevant information (Abujabal *et al.* 2018). This capability strengthens the reasoning process, providing a robust foundation for the LLM’s strategic planning while reducing the likelihood of hallucinations (Wei *et al.* 2022).

**Figure 1.**
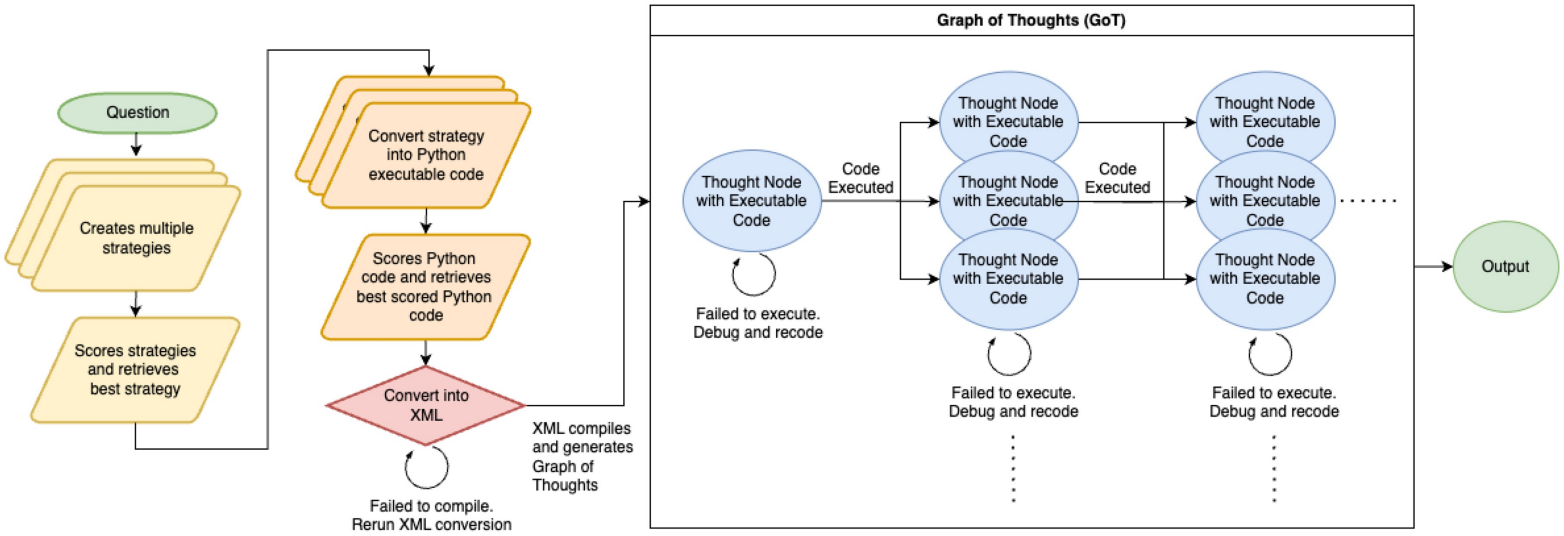
Algorithm flow chart (above) describing ESCARGOT’s approach to strategize, create python executable code, convert to machine readable XML code, deploy the Graph of Thoughts, and return the output.

## 2 Methodology

In this section, we outline the key components and processes underlying ESCARGOT’s design. We will detail ESCARGOT’s workflow, describing how ESCARGOT integrates LLMs with knowledge graphs to improve accuracy and contextual relevance. We will also explain how Graph of Thoughts are generated by the initial query, and detail the querying mechanisms via Cypher queries and Retrieval-Augmented Generation (RAG), which allow ESCARGOT to leverage structured, factual information from knowledge graphs. This methodology provides a novel framework for reducing hallucinations and supporting more advanced reasoning in LLMs.

### 2.1 ESCARGOT workflow

The ESCARGOT system is designed to integrate large language models (LLMs) with knowledge graphs to enhance reasoning capabilities and minimize hallucinations. It achieves this through a structured process that combines strategy generation, code execution, and knowledge retrieval (Figure 1). The following steps outline the workflow:

1. Strategy generation: ESCARGOT begins by generating multiple strategies in response to a user query or task. Using the LLM, these strategies incorporate relevant relationships and node types from the underlying knowledge graph. By default, three distinct strategies are created, each offering different approaches to solving the problem.
2. Strategy scoring and assessment: The system evaluates and scores each of the generated strategies based on several criteria, including clarity, comprehensiveness, and alignment with the schema of the knowledge graph. This scoring process is essential in determining which strategy is most likely to yield accurate and relevant results. The strategy with the highest score is then selected for further processing.
3. Python code generation: After selecting the optimal strategy, ESCARGOT converts it into executable Python code. The generated code is designed to utilize predefined functions and libraries, ensuring compatibility with the ESCARGOT framework. This step involves translating the high-level strategy into detailed, executable instructions that the system can run efficiently.
4. XML conversion and compilation: Once the Python code is generated, it is transformed into an XML format, which serves as the structural backbone of the Graph of Thoughts (GoT). The XML defines a machine-readable representation of the reasoning process, where each node corresponds to a specific code execution task, and edges represent the logical flow between tasks. If the XML fails to compile correctly, the system returns to the strategy generation phase to refine the conversion process.
5. GoT Python execution framework: The GoT framework dynamically organizes the code execution into interconnected nodes, with each node containing a piece of Python code ready for execution. ESCARGOT traverses the GoT, executing each node sequentially. If any node fails to execute, the system enters a self-debugging loop, where the error is analyzed, the code is revised, and the node is re-executed. This ensures the GoT remains adaptable and resilient, dynamically adjusting to errors and improving its execution with each iteration. This approach mitigates the risk of overloading the LLM with lengthy or confusing contexts during the knowledge extraction process (Chen *et al.* 2023). By converting knowledge into interactable Python objects using Cypher or RAG and performing operations on them directly, ESCARGOT preserves the fidelity of information and enhances accuracy. The Python objects generated at each node are passed as pre-loaded variables to subsequent nodes within the graph’s code environment.
  Direct Python execution is especially beneficial for tasks requiring precision, such as performing an intersection between two arrays, where it clearly outperforms LLMs on large arrays. This method is not only faster but also reduces hallucinations and errors in complex tasks. Computational operations are deterministic and reliable, minimizing the risk of mistakes. The results are stored and utilized by subsequent nodes within the GoT framework.
  The added complexity brought by executing any possible Python code, ESCARGOT must have the capability for self-debugging and generating new code if the initial code fails to compile (Madaan *et al.* 2024). If an error is detected during execution, the system can analyze the issue, revise the code, and reattempt compilation autonomously. This feature ensures resilience and adaptability in code execution, reducing the need for manual intervention and improving the overall efficiency and reliability of the system.
6. Knowledge retrieval integration: To support accurate execution and reduce the risk of hallucinations, ESCARGOT integrates two forms of knowledge retrieval:
  - Cypher queries: These queries interact with a knowledge graph (e.g. Memgraph, Neo4j), retrieving structured, factual information based on relationships between entities such as genes, diseases, or drugs. The GoT framework dynamically generates Cypher queries to extract the most relevant data, guiding the reasoning process in real time (Quamar *et al.* 2022). By default, ESCARGOT prefers Cypher queries over vector databases, as Cypher queries provide more exact knowledge extraction without an upper limit on the amount of knowledge retrieved.
  - Vector database requests: As a backup to Cypher queries, ESCARGOT can also perform similarity searches using vector databases. The GoT is embedded into a vector space to retrieve contextually relevant information based on vector similarity (Dilocker *et al.*). While this method supports the ongoing reasoning process, it is primarily used when Cypher queries are unavailable or insufficient due to their token limits.
7. Output and result generation: After successful execution of the GoT, the results are compiled and delivered to the user as an output. Each step of the reasoning process is documented, ensuring transparency in the decision-making process, while the use of knowledge graphs ensures that the conclusions are grounded in factual data.

## 3 Benchmarking and results

To demonstrate ESCARGOT’s practicality and versatility, we applied it to a publicly available Alzheimer’s knowledge graph, AlzKB (https://alzkb.ai/). AlzKB is a Memgraph graph database containing detailed data on genes, diseases, drugs, and other relevant entities, along with the semantic relationships between them (Romano *et al.* 2023). We will be using the 1.21 version of AlzKB for our benchmarks. We prepared a Weaviate vector database, which holds descriptions of all nodes and relationships from AlzKB for vectorized retrieval.

### 3.1 Dataset preparation

We evaluated ESCARGOT using six datasets designed to stress the capabilities of an LLM interacting with a knowledge graph. These datasets feature 1-hop and 2-hop reasoning across three types of questions: multiple choice, true/false, and open-ended. Each question is hand designed with a natural language question with respective Cypher queries in mind, testing ESCARGOT’s flexibility in reasoning by comparing its answers against ground truth data.

### 3.2 Query processing and response generation

When a user poses a query, such as the following open-ended question, “List the body parts/anatomy which over-express the genes METTL5 and STYXL2,” ESCARGOT processes the question by first analyzing its context through the LLM. ESCARGOT then generates multiple strategies and code snippets to solve the problem, constructing a graph of executable steps. For the above query, ESCARGOT might generate the following strategy:

1. Find the body parts or anatomy that over-expresses the gene METTL5.
2. Find the body parts or anatomy that over-expresses the gene STYXL2.
3. List the intersection of body parts from steps 1 and 2.

ESCARGOT links these steps into a graph structure, where each node corresponds to a specific computation or Cypher query, and the edges define the flow of information between nodes. Python code is generated for each node, ensuring proper execution. The variables defined and created within a node’s code will be passed to the next accordingly. If any issues arise during code execution or knowledge retrieval, ESCARGOT’s debugging mechanisms adaptively refine the code or queries for improved results. Once all nodes are executed without fail, ESCARGOT will synthesize the flow of information into a final node, providing an answer to the user.

### 3.3 Results

To evaluate ESCARGOT’s performance, we used OpenAI’s GPT-4o Mini as our baseline LLM due to its affordability and competitive performance relative to current open-source models. While we expect that performance would improve with more advanced models, GPT-4o Mini provides a reasonable benchmark. We implemented three methods for comparison: a vanilla input/output (I/O) approach, a standard Retrieval-Augmented Generation (RAG) implementation using a vector database, and our ESCARGOT package. The output of each method was compared against ground truth data. We score the response based on an exact match with the ground truth for true/false and multiple choice questions and for how much overlap for open-ended questions.

ESCARGOT consistently outperformed the other methods, particularly in open-ended questions, where it avoided the context length limitations inherent to RAG systems (Table 1). By encapsulating the entire knowledge retrieval process within Python objects, ESCARGOT maintained full knowledge fidelity, leading to superior results. This advantage was also apparent in the True/False and Multiple Choice questions where it achieved similar to better results. Detailed experiments and full results are available in our GitHub repository.

## 4 Conclusion

In this paper, we introduced ESCARGOT, a novel AI agent framework designed to mitigate hallucinations in LLMs by integrating dynamic reasoning through the Graph of Thoughts (GoT) framework with robust knowledge retrieval mechanisms. ESCARGOT dynamically generates a GoT based on specific queries, informed by the LLM’s strategy formulation, and executes this plan through a Python-based AI agent. By leveraging Cypher-based extraction from knowledge graphs and vector database queries, ESCARGOT ensures the retrieval of relevant, contextually accurate information while minimizing hallucinations. ESCARGOT’s support for direct Python execution of code snippets allows the AI agent to handle complex computational tasks, offering a reliable method for tasks like array operations and beyond.

**Table 1.** Knowledge graph datasets and methods.

Dataset	GPT-4o Mini (%)	RAG (%)	ESCARGOT (%)
Openended 1-hop (508 questions)	4.2	55.5	**88.4**
Openended 2-hop (450 questions)	4.9	23.1	**85.8**
True/false 1-hop (560 questions)	60.5	85.2	**90.9**
True/false 2-hop (540 questions)	59.4	**75.6**	74.1
Multiple choice 1-hop (498 questions)	58.2	88.8	**93.4**
Multiple choice 2-hop (419 questions)	61.3	86.4	**89.5**

Bold indicates highest values.

ESCARGOT is generalizable to any knowledge graph, and we demonstrated ESCARGOT’s capabilities with real-world examples, showcasing its ability to retrieve precise information, generate coherent, natural responses, and explain the reasoning behind each decision. This makes ESCARGOT a valuable AI agent for researchers and practitioners seeking high accuracy and reliability, particularly in critical fields such as healthcare where transparent and trustworthy reasoning is paramount.

Future advancements could focus on fine-tuning specialized models for different components of the ESCARGOT AI agent pipeline. For instance, distinct models could be trained for strategy generation, Cypher query formulation, and knowledge aggregation, enhancing both the system’s accuracy and its efficiency in solving complex, multi-step problems. Given that our benchmarking used OpenAI’s GPT-4o Mini, a model of similar or slightly lower performance than top open-source alternatives, there is potential to implement ESCARGOT entirely locally, enabling efficient, cost-effective deployment with no data leakage.

ESCARGOT also sets the stage for future research and development in AI agent technology, especially in interactive applications that leverage language models and knowledge graphs. Its application in the medical domain is especially promising, where doctors could integrate patient data and biomedical resources into the system. ESCARGOT’s dynamic reasoning framework, coupled with AI agent transparency, could help clinicians navigate complex medical challenges and deliver personalized, evidence-based solutions with clear, explainable reasoning.
